# Supplementary material for: The Effect of Dietary Patterns on Inflammatory Biomarkers in Adults with Type 2 Diabetes Mellitus: A Systematic Review and Meta-Analysis of Randomized Controlled Trials
Source: Nutrients. 2022 Oct 31;14(21):4577. doi: 10.3390/nu14214577 (PMC9654560; doi:10.3390/nu14214577)
Supplement: Supplementary file 1 [file nutrients-14-04577-s001.zip › nutrients-1917274-supplementary.pdf]

Supplementary Materials for peer-review only

Supplemental Tables * For review

Sensitivity analyses/subgroups

Table S1. Subgroup of Control Diet.

Subgroup	Low fat	Habitual Diet	Same Diet of experimental + Placebo	Other Type of diet
Control Diet	- Maiorino Maria Ida<br>- Lasa, A.<br>- Golan Rachel<br>- Fernemark Hanna	- Itsiopoulos C.	- Sauder, Katherine<br>- Medina Vera I	- Dylan Thompson<br>- Azadbakht Leila<br>- L. Maria Belalcazar

Low- fat: Adiponectin Heterogeneity: $Tau^2 = 0.19$; $Chi^2 = 10.35$, df = 2 ($P = 0.006$); $I^2 = 81\%$.
PCR; Other Type of diet: Adiponectin; Heterogeneity: $Tau^2 = 0.71$; $Chi^2 = 84.66$, df = 1 ($P < 0.00001$); $I^2 = 99\%$.

Table 2. Subgroup of type of center.

Subgroup	Single Center	Multicenter
Center	- Azadbakht Leila<br>- Fernemark Hanna<br>- Itsiopoulos C.<br>- Golan Rachel<br>- Maiorino Maria Ida<br>- Medina Vera I<br>- Sauder, Katherine	- Dylan Thomson<br>- L. Maria Belalcazar<br>- Lasa, A.

Single Center: Adiponectin;Heterogeneity: $Tau^2 = 0.39$; $Chi^2 = 5.53$, df = 1 ($P = 0.02$); $I^2 = 82\%$,.
PCR; Heterogeneity: $Tau^2 = 0.91$; $Chi^2 = 53.87$, df = 4 ($P < 0.00001$); $I^2 = 93\%$.
Multicenter: Adiponectin;Heterogeneity: $Tau^2 = 0.51$; $Chi^2 = 85.64$, df = 2 ($P < 0.00001$); $I^2 = 98\%$,.
PCR;Heterogeneity: $Tau^2 = 0.36$; $Chi^2 = 44.92$, df = 1 ($P < 0.00001$); $I^2 = 98\%$.

Table S3. Subgroup of follow-up.

Subgroup	< 24 weeks	≥ 52 weeks
Follow-up	- Itsiopoulos C.<br>- Sauder, Katherine<br>- Medina Vera<br>- Azadbakht Leila<br>- Fernemark Hanna	- Dylan Thompson<br>- Maiorino Maria Ida<br>- Lasa, A.<br>- Golan Rachel<br>- L. Maria Belalcazar

PCR < 52 weeks: Heterogeneity: $Tau^2 = 1.93$; $Chi^2 = 41.81$, df = 2 ($P < 0.00001$); $I^2 = 95\%$,.
≥ 52 weeks Heterogeneity: $Tau^2 = 0.26$; $Chi^2 = 55.52$, df = 3 ($P < 0.00001$); $I^2 = 95\%$.
Adiponectin: All of studies are ≥ 52 weeks Heterogeneity: $Tau^2 = 0.38$; $Chi^2 = 96.28$, df = 4 ($P < 0.00001$); $I^2 = 96\%$.

Table S4. Subgroup of Control Glycemic.

Subgroup	HbA1c (%)<br>< 7.0<br>Or/ and<br>Fasting plasma glucose (mg/dl) 70–130	HbA1c (%)<br>> 7.0<br>Or/ and<br>Fasting plasma glucose (mg/dl) > 130
Control Glycemic	- Dylan Thompson<br>- Sauder, Katherine	- Maiorino Maria Ida<br>- Lasa, A.<br>- Itsiopoulos C.<br>- Medina Vera I<br>- Golan Rachel<br>- L. Maria Belalcazar<br>- Fernemark Hanna

*Azadbakht Leila: did not reported values.
< 7.0 % HbA1c, PCR: Heterogeneity: $Tau^2 = 0.63$; $Chi^2 = 14.62$, df = 1 (P = 0.0001); $I^2 = 93\%$.
> 7.0 % HbA1c, Adiponectin: Heterogeneity: $Tau^2 = 0.11$; $Chi^2 = 17.70$, df = 3 (P = 0.0005); $I^2 = 83\%$.
PCR: Heterogeneity: $Tau^2 = 0.24$; $Chi^2 = 58.47$, df = 4 (P < 0.00001); $I^2 = 93\%$.

Table S5. Subgroup of Region.

Subgroup	The Americas	Europe	Oceania	Asia
**Region**	- Look AHEAD L. Maria Belalcazar (2012)<br>- Sauder, Katherine (2015)<br>- Medina Vera I (2018)	- ACTID Dylan Thompson (2014)<br>- MÉDITA Maiorino Maria Ida (2016)<br>- Fernemark Hanna (2013)<br>- PREDIMED Lasa, A. (2014)	- Itsiopoulos C. (2011)	- DIRECT Golan Rachel (2011)<br>- Azadbakht Leila (2011)

Region Europe: (lower in Adiponectin) Heterogeneity: $Tau^2 = 0.37$; $Chi^2 = 35.40$, df = 2 (P < 0.00001); $I^2 = 94\%$, (before 96%) in CPR is highest (97%).
In Asia (98%). Region the Americas (Look AHEAD and Sauder, Katherine (2015) heterogeneity was lower in PCR. Heterogeneity: $Tau^2 = 0.00$; $Chi^2 = 1.08$,.
df = 1 (P = 0.30); $I^2 = 8\%$.

Table S6. Subgroup of Study Design.

Subgroup	Control vs Experimental study	Cross-over study
Study Design	- Dylan Thomson<br>- Golan Rachel<br>- L. Maria Belalcazar<br>- Lasa, A.<br>- Maiorino Maria Ida<br>- Medina Vera I	- Azadbakht Leila<br>- Fernemark Hanna<br>- Itsiopoulos C.<br>- Sauder, Katherine

All studies evaluated Adiponectin were Control vs experimental. Control vs experimental (PCR) Heterogeneity: $Tau^2 = 0.26$; $Chi^2 = 55.52$,.
df = 3 (P < 0.00001); $I^2 = 95\%$ Cross-over: Heterogeneity: $Tau^2 = 1.93$; $Chi^2 = 41.81$, df = 2 (P < 0.00001); $I^2 = 95\%$.

Table S7. Subgroup of Type of Diet.

Subgroup	Prescribed	Recommendations
Type of diet (Med Diet)	- Maiorino Maria Ida<br>- Itsiopoulos C.<br>- Sauder, Katherine<br>- Medina Vera I<br>- Golan Rachel<br>- L. Maria Belalcazar<br>- Fernemark Hanna	- Dylan Thompson<br>- Lasa, A.<br>- Azadbakht Leila

Prescribed (adiponectin): Heterogeneity: $Tau^2 = 0.17$; $Chi^2 = 17.61$, df = 2 (P = 0.0001); $I^2 = 89\%$ / Recommendations (adiponectin): Heterogeneity: $Tau^2 = 0.69$; $Chi^2 = 28.80$, df = 1 (P < 0.00001); $I^2 = 97\%$.Prescribed (PCR): Heterogeneity: $Tau^2 = 0.08$; $Chi^2 = 15.46$, df = 4 (P = 0.004); $I^2 = 74\%$ / Recommentations (PCR); Heterogeneity: $Tau^2 = 5.75$; $Chi^2 = 66.20$, df = 1 (P < 0.00001); $I^2 = 98\%$.

Table S8. Subgroup of Control and Experimental Diet.

Subgroup	Study
Med Diet vs Low fat	- Lasa<br>- Maiorino<br>- Golan Rachel

(Adiponectin) Heterogeneity: $Tau^2 = 0.19$; $Chi^2 = 10.35$, df = 2 (P = 0.006); $I^2 = 81\%$.
(PCR: Heterogeneity): $Tau^2 = 0.76$; $Chi^2 = 9.69$, df = 1 (P = 0.002); $I^2 = 90\%$.

Table S9. Subgroup of Med Diet.

Subgroup	Study (PCR)
Med Diet	- Itsiopoulos C.<br>- Maiorino<br>- Golan Rachel

PCR: Heterogeneity: $Tau^2 = 0.38$; $Chi^2 = 13.54$, df = 2 (P = 0.001); $I^2 = 85\%$.

Data for meta-analysis *For review

ADIPONECTIN (ug/mL)

**Study**	**Diet control**<br>**Basal measurement**<br>**Final measurement**<br>**N**<br>**Mean (SD)**	**Diet experimental**<br>**Basal measurement**<br>**Final measurement**<br>**N**<br>**Mean (SD)**
ACTID<br>**Dylan Thompson**	5.0 (1.7)<br>5.50<br>99	5.0 (1.7)<br>5.79<br>248
MÉDITA trial<br>**Maiorino Maria Ida**	6.442 (1.57)<br>7.10 (1.7)<br>97	6.17 (1.67)<br>8.87 (1.9)<br>102
PREDIMED<br>**Lasa, A.**	1.58 (6.3)<br>3.1 (3.9)<br>67	5.011 (5.011)<br>7.9 (3.1)<br>74
**LOOK AHEAD**	5.025 (1.03)	4.75 (0.97)

	L. Maria Belalcazar	2.42 (0.108)<br>836	2.87 (0.49)<br>922
	DIRECT<br>**Golan Rachel**	7.3 (2.6)<br>12	7.3 (2.8)<br>15
	**Study**	**Diet control**<br>**Basal measurement**<br>**Final measurement**<br>**N**<br>**Mean (SD)**	**Diet experimental**<br>**Basal measurement**<br>**Final measurement**<br><br>**Mean (SD)**
**CRP** (mg/L)	ACTID<br>**Dylan Thompson**	2.3 (3.4)<br>1.53<br>99	2.0 (3.0)<br>1.68<br>248
	MÉDITA trial<br>**Maiorino Maria Ida**	3.1 (1.04)<br>2.84 (1.01)<br>97	3.0 (0.93)<br>1.94 (0.78)<br>102
	**Itsiopoulos C.**	-<br>2.49 (0.50)<br>27	-<br>2.38 (0.46)<br>27
	**Sauder, Katherine**	1.73 (0.53)<br>2.16 (0.16)<br>30	1.73 (0.53)<br>1.98 (0.16)<br>30
	DIRECT<br>**Golan Rachel**	4.1 (3.2)<br>3.6 (2.9)<br>12	5.0 (3.4)<br>4.6 (3.4)<br>15
	**Azadbakht Leila**	3.11 (0.30)<br>2.91 (0.30)<br>31	2.90 (0.31)<br>2.04 (0.20)<br>31

PRISMA 2020 Checklist. *For review

Section and Topic	Item #	Checklist item	Location where item is reported
**TITLE**			
Title	1	Identify the report as a systematic review.	Page 1
**ABSTRACT**			
Abstract	2	See the PRISMA 2020 for Abstracts checklist.	Page 1
**INTRODUCTION**			
Rationale	3	Describe the rationale for the review in the context of existing knowledge.	Page 1,2
Objectives	4	Provide an explicit statement of the objective(s) or question(s) the review addresses.	Page 2
**METHODS**			
Eligibility criteria	5	Specify the inclusion and exclusion criteria for the review and how studies were grouped for the syntheses.	Page 3
Information sources	6	Specify all databases, registers, websites, organisations, reference lists and other sources searched or consulted to identify studies. Specify the date when each source was last searched or consulted.	Page 2
Search strategy	7	Present the full search strategies for all databases, registers and websites, including any filters and limits used.	Page 2
Selection process	8	Specify the methods used to decide whether a study met the inclusion criteria of the review, including how many reviewers screened each record and each report retrieved, whether they worked independently, and if applicable, details of automation tools used in the process.	Page 3,4
Data collection process	9	Specify the methods used to collect data from reports, including how many reviewers collected data from each report, whether they worked independently, any processes for obtaining or confirming data from study investigators, and if applicable, details of automation tools used in the process.	Page 4

Section and Topic	Item #	Checklist item	Location where item is reported
Data items	10a	List and define all outcomes for which data were sought. Specify whether all results that were compatible with each outcome domain in each study were sought (e.g. for all measures, time points, analyses), and if not, the methods used to decide which results to collect.	Page 4,5
	10b	List and define all other variables for which data were sought (e.g. participant and intervention characteristics, funding sources). Describe any assumptions made about any missing or unclear information.	Page 4
Study risk of bias assessment	11	Specify the methods used to assess risk of bias in the included studies, including details of the tool(s) used, how many reviewers assessed each study and whether they worked independently, and if applicable, details of automation tools used in the process.	Page 4
Effect measures	12	Specify for each outcome the effect measure(s) (e.g. risk ratio, mean difference) used in the synthesis or presentation of results.	Page 4,5
Synthesis methods	13a	Describe the processes used to decide which studies were eligible for each synthesis (e.g. tabulating the study intervention characteristics and comparing against the planned groups for each synthesis (item #5)).	Page 4, 5
	13b	Describe any methods required to prepare the data for presentation or synthesis, such as handling of missing summary statistics, or data conversions.	Page 5
	13c	Describe any methods used to tabulate or visually display results of individual studies and syntheses.	Page 5
	13d	Describe any methods used to synthesize results and provide a rationale for the choice(s). If meta-analysis was performed, describe the model(s), method(s) to identify the presence and extent of statistical heterogeneity, and software package(s) used.	Page 5
	13e	Describe any methods used to explore possible causes of heterogeneity among study results (e.g. subgroup analysis, meta-regression).	Page 5
	13f	Describe any sensitivity analyses conducted to assess robustness of the synthesized results.	Page 5
Reporting bias assessment	14	Describe any methods used to assess risk of bias due to missing results in a synthesis (arising from reporting biases).	Page 4,5
Certainty assessment	15	Describe any methods used to assess certainty (or confidence) in the body of evidence for an outcome.	Page 4
**RESULTS**			

Section and Topic	Item #	Checklist item	Location where item is reported
Study selection	16a	Describe the results of the search and selection process, from the number of records identified in the search to the number of studies included in the review, ideally using a flow diagram.	Page 6
	16b	Cite studies that might appear to meet the inclusion criteria, but which were excluded, and explain why they were excluded.	Page 6
Study characteristics	17	Cite each included study and present its characteristics.	Page 6 y appendix A1
Risk of bias in studies	18	Present assessments of risk of bias for each included study.	Page 8
Results of individual studies	19	For all outcomes, present, for each study: (a) summary statistics for each group (where appropriate) and (b) an effect estimate and its precision (e.g. confidence/credible interval), ideally using structured tables or plots.	Page 7
Results of syntheses	20a	For each synthesis, briefly summarise the characteristics and risk of bias among contributing studies.	Page 7
	20b	Present results of all statistical syntheses conducted. If meta-analysis was done, present for each the summary estimate and its precision (e.g. confidence/credible interval) and measures of statistical heterogeneity. If comparing groups, describe the direction of the effect.	Page 12
	20c	Present results of all investigations of possible causes of heterogeneity among study results.	Page 13
	20d	Present results of all sensitivity analyses conducted to assess the robustness of the synthesized results.	Supple,M
Reporting biases	21	Present assessments of risk of bias due to missing results (arising from reporting biases) for each synthesis assessed.	Supple.M.
Certainty of evidence	22	Present assessments of certainty (or confidence) in the body of evidence for each outcome assessed.	Page 12
**DISCUSSION**			
Discussion	23a	Provide a general interpretation of the results in the context of other evidence.	Page 14

**Section and Topic**	**Item #**	**Checklist item**	**Location where item is reported**
	23b	Discuss any limitations of the evidence included in the review.	Page 15
	23c	Discuss any limitations of the review processes used.	Page 15
	23d	Discuss implications of the results for practice, policy, and future research.	Page 15
**OTHER INFORMATION**			
Registration and protocol	24a	Provide registration information for the review, including register name and registration number, or state that the review was not registered.	Page 2
	24b	Indicate where the review protocol can be accessed, or state that a protocol was not prepared.	Page 2
	24c	Describe and explain any amendments to information provided at registration or in the protocol.	Page 2
Support	25	Describe sources of financial or non-financial support for the review, and the role of the funders or sponsors in the review.	Page 16
Competing interests	26	Declare any competing interests of review authors.	Page 16
Availability of data, code and other materials	27	Report which of the following are publicly available and where they can be found: template data collection forms; data extracted from included studies; data used for all analyses; analytic code; any other materials used in the review.	Suppl. Mat.

The "Risk of Bias" tool. Clinical Trials.

Table S10. The "Risk of Bias" tool. Clinical Trials. *For review.

**Rubro/ Art**	**Dylan Thompson**	**Saunder Katherine**	**Maiorino Maria Ida**	**Lasa, A.**	**Itsiopoulos C.**	**Medina Vera**	**Golan Rachel**	**Azadbakht Leila**	**L. Maria Belalcazar**	**Fernemark Hanna**
**Selection** Sequence generation	**Low risk** Quote: "Patients were assigned using a computer-generated allocation in a 2:5:5 ratio to usual care, an intensive diet intervention, or the intensive diet intervention plus activity."	**Low risk** Quote: "Test diets were presented in a counterbalanced order for 4 weeks each, with order determined by simple randomization and equal allocation to each treatment order assignment"	**Low risk** Quote:"Randomization started in 2004, and the trial ended in early fall 2012, after a total follow-up of 8.1 years."	**Low risk** Quote: "Study participants were randomized to three equally sized groups".	**Low risk** Quote "Subjects were randomized to commence."	**Low risk** Quote: "At the third visit, all subjects were randomized to receive either the DP or P treatment in combination with the reduced energy diet for 1 month."	**Low risk** Quote:" we randomly assigned 322 moderately obese subjects "	**Low risk** Quote: "We used a randomized cross-over design. After a 3-wk run-in, patients were randomly assigned to 8 wk of a control diet or the DASH diet."	**Low risk** Quote:"Eligible participants are randomly assigned to either diabetes support and education or lifestyle intervention using a web-based data management system that verifies eligibility."	**Low risk** Quote: "Each patient tested all the three different diets in randomized order."
**Selection** Allocation sequence concealment	**Low risk** Quote:"Allocation was stratified by center and minimized by age, sex, fitness, route into the study, and blood pressure."	**Low risk** Quote: "The study coordinator generated the random allocation sequence and assigned participants to the interventions."	**Low risk** Quote: "The two-arm trial design it was in equilibrium and allocation was in sealed study folders".	**Low risk** Quote: "Tables of random allocation were centrally elaborated."	**Unclear risk** Quote: "To commence with either the intervention diet or the control diet (habitual) for 12 weeks and then cross over to the alternate diet for the	**Unclear risk** Comment: Not describe allocation method.	**Low risk** Quote: "Strata of sex, age (below or above the median), BMI (below or above the median), history of coronary heart disease (yes or no), history of type 2 diabetes (yes or no), and	**Low risk** Quote: "The project dietitian enrolled participants and randomly allocated them to groups using random sequencing generated in SPSS at the end of the run-in period."	**Low risk** Quote:" Randomization is stratified by clinical center and blocked with random block sizes."	**Low risk** Quote: "Randomization was performed by drawing ballots and the sequence of the diets was determined at one single occasion for all participants."

					following 12 weeks." Comment: Not specification about the procedure.		current use of statins (none, <1 year, or ≥1 year) with the use of Monte Carlo simulations."			
**Performance** Blinding of participants, personnel	**Low risk** Quote: "Dieticians, nurses, and patients were aware of allocation, but doctors were not. All assessments were performed by nurses" Quote: "Any changes in treatment of these features were made by a doctor unaware of treatment allocation and according to a strict trial protocol to keep the risk of performance bias to a minimum."	**High risk** Quote:" Due to the nature of the dietary intervention, metabolic kitchen staff and participants were aware of treatment assignments."	**High risk** Quote: "Who assessed achievement of the primary outcome were blinded to the intervention." Comment: No double-blinding	**Low risk** Quote: "The study nurses in charge of the random allocation were independent of the nursing staff. At baseline, general practitioners were not informed of the allocation of participants." Quote: "group sessions are organized separately for each of the three intervention groups"	**High risk** Quote: "Subjects were consulted individually by an accredited Practising Dietitian". Comment: It not clear the blinding of participants and personnel.	**Low risk** Comment: Double blind	**High risk** **Quote: "**The dietitians met with their groups in weeks 1, 3, 5, and 7 and thereafter at 6-week intervals, for a total of 18 sessions of 90 minutes each." "Each food item was provided with a label showing the number of calories and the number of grams of carbohydrates, fat, and saturated fat, according to an analysis based	**High risk** **Quote:"**patients were not blinded."	**High risk** **Quote: "**Participants are prescribed a diet of self-selected foods for the first 2 weeks." Comment: Participants and dietitians were not blinded.	**High risk** **Quote: "**All study meals were consumed under supervision by the study organizers (HF and CJ) at the Diabetes ward of Motala Hospital.

							on the Israeli nutritional database. .”<br><br>Comment: It seems the dietitians and patients were not blinded.			
**Detection** Blinding of outcome assesment	**High risk** Quote: “The diet was not prescriptive; goals were negotiated individually with each participant. “	**Low risk** Quote: “technicians who measured outcome variables were blinded to the diet assignments.”	**High risk** Quote: “Assessment of dietary adherence was based on self-report, and patients may have had incentives to misrepresent their adherence.”<br><br>“The investigators responsible for starting antihyperglycemic drug treatment were not blinded to trial assignment, and differences in assessment of patient information and patient management related to knowledge of that assignment cannot be	**Low risk** Quote:”Biological markers of compliance are measured in random subsets of participants from the three arms of the trial.”<br><br>Quote: “Outcomes are ascertained on a yearly basis by a Clinical Events Committee whose members are blinded to the intervention group.”	**High risk** Quote: “Completed diaries were reviewed and verified by an practicing Dietitian, and incomplete records were discarded. “<br>“Dietary intake was monitored by a self-completed seven-day diet record (in household measures).”<br>Comment: there are not evidence about blinding	**High risk** Quote: “Compliance with the dietary portfolio (DP) or placebo (P) intervention was evaluated by the number of empty packets returned the following visit.”<br><br>Comment: It seems to evaluator of intervention no was blinded.	**Low risk** Quote: “The clinic and laboratory staff members were unaware of the treatment assignments, and the study coordinators were unaware of all outcome data until the end of the intervention.”	**Low risk** Quote: “The laboratory staff was blinded to the treatment status.”	**Low risk** **Quote: “**personnel who are blinded to treatment assignment.”	**High risk** Comment: Open label

			totally excluded as contributing to the findings."		outcome assessment, it seems like the results it depended to answers de participants.					
**Attrition** Incomplete outcome data	**High risk** Quote: "Approximately 90% of potential blood samples were available for assessment of CRP, IL-6, adiponectin, and sICAM-1." Comment: Lost to follow up.	**High risk** Comment: 12%patients lost to follow up.	**Low risk** Quote: "The overall proportion of participants who were lost to follow-up was similar in both groups (9.2–9.3 %, respectively)"	**High risk** Quote: "The attrition rate after 2 years' follow-up for participants recruited before 2006 (n = 4.381) was 9.3% The study was not a randomized one and the sample size for each dietary intervention could be greater". Comment: Not explain causes or equilibrium for the losses.	**Low risk** Quote: "Twenty-seven adults (sixteen men and eleven women) completed the study" Comment: 100% of subjects completed the trial.	**High risk** Comment: Lost of follow up it was 34.5%, 16 in experimental group and 12 in placebo group. Authors do not give details.	**Unclear risk** Quote: " 84.6% compliance" Comment: It is not reported how it was equilibrium to loss of follow up.	**High risk** Quote: "Eleven patients did not follow the study protocol)." Comment: 29% subjects lost to follow up.	**Low risk** Quote: "Two percen to the participantsare lost annually with respect to end point ascertainment in each arm." Quote: "the planned length of follow-up is not fully achieved."	**Low risk** Quote: "The remaining 19 participants completed all three experimental." "compliance was assumed to be excellent and hence to give high statistical power, but due to risk of attrition, we planned to include about 20 subjects"
**Reporting** Selective outcome reporting	**Low risk** Quote: "Results demonstrate that motivational, unsupervised diet and diet plus physical activity interventions integrated	**Low risk** Comment: In order to objective, give it results, and conclusion.	**Low risk** Quote: "The analysis of CRP and adiponectin was not the primary end point of the MÉDITA trial, which in	**Unclear risk** Quote: "the primary aim cardiovascular disease prevention. The major strength is that a wide panel of	**High risk** Comment: changes about the primary outcome of the	**High risk** Comment: It is not reported basal measurements of PCR.	**Low risk** Quote: "Intercorrelations can be documented among markers of inflammation,	**Low risk** Quote: "we assess the effects of DASH on novel cardiovascular risk factors such	**High risk** Quote: "The ILI and DSE interventions have been described previously and are only briefly summarized here."	**Low risk** Quote: "All 19 patients with type 2 diabetes tested the three different

	into real-world healthcare settings generate beneficial changes in various markers of inflammation in patients with newly diagnosed T2D."<br>Comment: Objective, results, and conclusion are congruent.		theory could have resulted in group imbalance; however, the groups were well compa- rable for many baseline characteristics, including inflam- matory markers."	adipokines and related molecules involved in glucose metabolism was measured."<br>"Taking together, the results obtained in the present study show that Mediterranean diets, supplemented with virgin olive oil or nuts, are as efficient as a low-fat diet for reducing abdominal fat and improving parameters related to glycaemic control. "<br>Comment: T this study had a post hoc analysis, the investigators do not explain under which analysis (glycaemic control or cardiovascular disease prevention).	study, but basal data were not report.	Just give us the final difference.	hepatic steatosis, glucose homeostasis, and lipid profile"<br><br>Comment: objective and results are accurate.	as CRP, coagulation abnormalities, and tests of hepatic function among type 2 diabetic patients."<br>Comment: Reporting of outcome results were observed.	Comment: multiple reports could be confused.	diets for breakfast and lunch"<br><br>Comment: All of results were reported-
**Other bias**	**High risk**<br><br>Disclosures<br>Quote: "Thompson has acted as a consultant for Unilever. Andrews has	**High risk**<br>Statistical Analyses<br>Quote: "The present results should be	**High risk**<br><br>Follow up<br><br>Quote:	**High risk**<br><br>Genetic predisposition	**High risk**<br><br>Enrollment<br><br>Quote: "Volunteers with well-	**Unclear risk**<br><br>Disclosures<br>Quote: "Isabel Medina received a scholarship from	**High risk**<br><br>Equilibrium in the arms	**Low risk**<br><br>Treatment on DM2	**Low risk**<br><br>Study design	**High risk**<br>Intervention<br><br>Quote: "patients returned to regular eating-habits

	received honoraria from GlaxoSmithKline, Novo Nordisk, Sanofi-Aventis, and Lilly as well as travel expenses from Sanofi-Aventis".<br>Comment: they have conflicts of interest to this publication.	interpreted cautiously as they are secondary analyses of a study designed and powered for a primary outcome of systemic hemodynamics."<br><br>Comment: Study design could be compromised.	"Randomization started in 2004, and the trial was completed after a follow-up of 4 years. Subsequently, we decided to continue to monitor the participants who did not reach the primary end point. This report consists of the data obtained until 30 September 2012 (i.e., when the last patient reached the primary end point), with a total follow-up of 8.1 years."	Quote: "Genetic influences on our data cannot be excluded."	controlled type 2 diabetes were invited to participate via local newspaper advertisements."	CONACYT and Instituto Danone de México."<br><br>Comment: it is seeming to project sponsored.<br><br>**Unclear risk**<br><br>Enrollment<br>Quote:" were recruited through advertisements at the Department of Physiology of Nutrition at the Instituto Nacional de Ciencias Médicas y Nutrición Salvador Zubirán, Mexico City."<br><br>Comment: Are they patients and not patients in the enrollment?	Quote: "We enrolled few women; however, we observed a significant interaction between the effects of diet group and sex on weight loss (women tended to lose more weight on the Mediterranean diet), and this difference between men and women was also reflected in the changes in leptin levels."	Quote: "During the study, the physician was requested not to change medication dosages to avoid any resulting bias. "	Quotes: "Is the longest randomized controlled evaluation to date."<br>"lifestyle intervention was effective over 8 years in both men and women and across an ethnically and racially diverse population."<br><br>Comment: Is a good structure study.	in between the three trial days, and that we had no control of diets eaten on the days ahead of trial days."<br>High risk<br><br>Enrollment<br>Quote:"local advertising and by direct contact from the study organizers with potentially suitable patients."

Search Term of electronic of each electronic database

*****Coding**

("dietary pattern " OR "diet* pattern" OR "food pattern" OR "Mediterranean diet" OR "diet" or "Mediterranean" OR "Anti-inflammatory* Diet*" OR "score" OR "pattern" OR "adherence" OR "index" OR diet* index* OR dietary* patterns*" OR "eating pattern" OR "eating* patterns*" OR "food* pattern*" OR "food* patterns*" OR "dietary* habit*" OR "feeding* behaviour*") AND ("adult" OR "adults" OR "adulthood" OR "men" OR "man" OR "women" OR "woman") in combination with ("biomarker*" OR "bio-marker" OR "inflammation" OR " inflammatory") OR "endothelial" OR "vascular" OR "anti-inflammatory" OR "anti-inflammatory biomarkers") OR ("C-reactive protein" OR "CRP" OR "hsCRP" OR "high sensitivity C-reactive protein") OR ("TNF-α" OR "tumor necrosis factor" OR "tumor necrosis factor" OR TNF) OR ("interleukin 6" OR "interleukin-6" OR "IL-6") OR ("IL-1β" OR interleukin-1β" OR "interleukin 1beta") OR ("intercellular adhesion molecule 1" OR "ICAM-1") OR ("vascular cell adhesion molecule 1" OR "VCAM-1") OR ("retinol binding protein 4" OR "RBP4" OR "leptin" OR "leptin: adiponectin" OR "leptin/adiponectin") OR ("resistin") OR ("adiponectin" OR "adiponectin total" OR "HMW" OR "high molecular weight adiponectin") OR ("IFN-γ" OR "gamma interferon") OR ("interleukin 4" OR "interleukin-4" OR " IL-4") OR ("interleukin 10" OR "interleukin-10" OR "IL-10") OR ("transforming growth factor-b" OR "TGF-b" OR "TGF-beta" OR "TGF-β") AND ("diabetes mellitus type 2" OR "type 2 diabetes mellitus").

MEDLINE

(((Dietary pattern) OR (Food pattern)) OR (Anti-inflammatory Diet)) AND (type 2 diabetes mellitus) (Mediterranean Diet) AND (Diabetes Mellitus 2) ("TNF-α" OR "tumor necrosis factor" OR "tumor necrosis factor" OR TNF) OR ("interleukin 6" OR "interleukin-6" OR "IL-6") OR ("IL-1β" OR interleukin-1β" OR "interleukin 1beta") OR ("intercellular adhesion molecule 1" OR "ICAM-1") OR ("vascular cell adhesion molecule 1" OR "VCAM-1") OR ("retinol binding protein 4" OR "RBP4" OR "leptin" OR "leptin: adiponectin" OR "leptin/adiponectin") OR ("resistin") OR ("adiponectin" OR "adiponectin total" OR "HMW" OR "high molecular weight adiponectin") OR ("IFN-γ" OR "gamma interferon")

SCOPUS

dietary pattern OR diet pattern OR food pattern OR mediterranean diet OR diet or Mediterranean OR Anti-inflammatory Diet OR score OR pattern OR adherence OR index OR diet index OR dietary patterns OR eating pattern OR eating patterns OR food pattern OR food patterns OR dietary habit OR feeding behavior AND adult OR adults OR adulthood OR men OR man OR women OR woman AND biomarker OR bio-marker OR inflammation OR inflammatory OR endothelial OR vascular OR anti-inflammatory OR anti-inflammatory biomarkers OR C-reactive protein OR CRP OR hsCRP OR high sensitivity C-reactive protein OR TNF-α OR tumor necrosis factor OR tumour necrosis factor OR TNF OR interleukin 6 OR interleukin-6 OR IL-6 AND diabetes mellitus type 2 OR type 2 diabetes
mellitus AND clinical trial AND (LIMIT-TO (DOCTYPE,"ar")) AND (LIMIT-TO (SRCTYPE,"j"))
mediterranean diet OR diet or Mediterranean OR Anti-inflammatory OR mediterranean diet OR diet or Mediterranean OR Anti-inflammatory Diet AND adult OR adults OR adulthood OR men OR man OR women OR woman AND biomarker OR bio-marker OR inflammationOR inflammatory AND diabetes mellitus type 2 OR type 2 diabetes mellitus AND (LIMIT-TO (SRCTYPE,"j")) AND (LIMIT-TO (DOCTYPE,"ar")) AND (LIMIT-TO (LANGUAGE,"English"))

Cochrane Central Register of Controlled Trials

Diabetes mellitus type 2 AND Mediterranean diet AND Clinical trial “eating* patterns*” OR “food* pattern*” OR “food* patterns*” OR “dietary* habit*” OR “feeding* behaviour*”) AND (“adult” OR “adults” OR “adulthood” OR “men” OR “man” OR “women” OR “woman”) in combination with (“biomarker*” OR “bio-marker” OR “inflammation” OR “ inflammatory”) OR “endothelial” OR “vascular” OR “anti-inflammatory” OR “anti-inflammatory biomarkers”) en Título Resumen Palabra clave - (Se han buscado variaciones de la palabra).

Table S11. Characteristics and properties of components of Mediterranean diet styles.

	**Components of the MD**	**MÉDITA trial Meditarrean diet scale Maiorino, 2016**	**PREDIMED Lasa, 2014**	**Traditional Cretan MD Itsiopoulos, 2011**	**DIRECT trial by recommendations of Willett and Skerrett Golan, 2012**	**Combined MD by Fernemark, 2013**
**Foods with beneficial effect reported**	Cereals	Whole grains	Whole grains	**Traditional** wholegrain bread & potatoes	Whole grains, brown rice, whole wheat pasta, oats. Sparingly: potatoes, refined grains, white rice, bread & pasta.	Wholegrain bread
	Non-caloric drinks	–	–	Greek coffee & herbal tea	Coffee with 1% fat milk	Black coffee
	Fruits & Nuts	Fruits & nuts	Fruits & nuts	Dried fruit & nuts large quantity of fruits (563 g/day)	Fruits & nuts: five to seven walnuts, <2 0 g or 5 units)	Fruits & cashew nuts
	Vegetables	Vegetables	Vegetables	Vegetables (691 g/day) green leafy vegetables (280 g/day)	Vegetables	Vegetables cucumber
	Legumes	Legumes	Legumes	Legumes	Seed, beans, and tofu	Legumes
	Fish & Seafood White meat	Fish –	Fish white meat	Fish & seafood (fried whitebait, fried calamari, and asparagus omelet)	2 fish meals poultry eggs	Salmon –
	Olive oil / MUFA: SFA ratio	30 to 50 g of olive oil. MUFA: SFA	50 mL extra virgin olive oil/day approximately 1L/week or 4 tablespoons/ day	Olive oil (75 mL/day) > 50% MUFA	30 to 45 g of olive oil	Olives (38g)
	Moderate alcohol con-sumption	Men 10 and 50 g/day	Moderate consumption of red wine	Moderate alcohol (4% of energy from red wine)	–	Red wine: J.P. Chenet Cabernet Syrah 14% alc. (200mL/1)
		Women 5-25 g/day		–	–	–
	Spices	–	Home-made sauce (tomato, onion, garlic, and spices) + olive oil	–	–	Red pepper
**Foods with no beneficial effect reported**	Meat	–	Red & processed meat	Red meat & eggs	Sparingly: red meat	Low in red meat, replacing beef and lamb

	Dairy	—	—	Milk	1–2 servings a day	—
	Fast food	—	Avoid	—	—	—
	Sweets	—	Avoid	—	Sparingly	—
	Pastry	—	Avoid	Pastries	—	—
	Sugar-sweetened beverages	—	Avoid	Cakes	Sparingly	—
	Butter	—	Avoid	—	Sparingly	Butter 10 g

Qualitative analysis from each study that reported a Mediterranean Dietary Pattern style and was synthesized by representative components. Classification: Foods with beneficial effect reported and Foods with no beneficial effect reported according to the American Diabetes Association [45], U.S. Government/National Academy of Sciences [67]. **Abbreviations**: DIRECT trial; Diabetes Remission Clinical Trial, MD; Mediterranean diet, MÉDITA trial; Mediterranean diet and type 2 diabetes, MD; Mediterranean Diet, MUFA; mono-unsaturated fatty acid, PUFA; polyunsaturated fatty acid, PREDIMED; Prevención con Dieta Mediterranean, SFA; saturated fatty acid.

Supplementary Figures

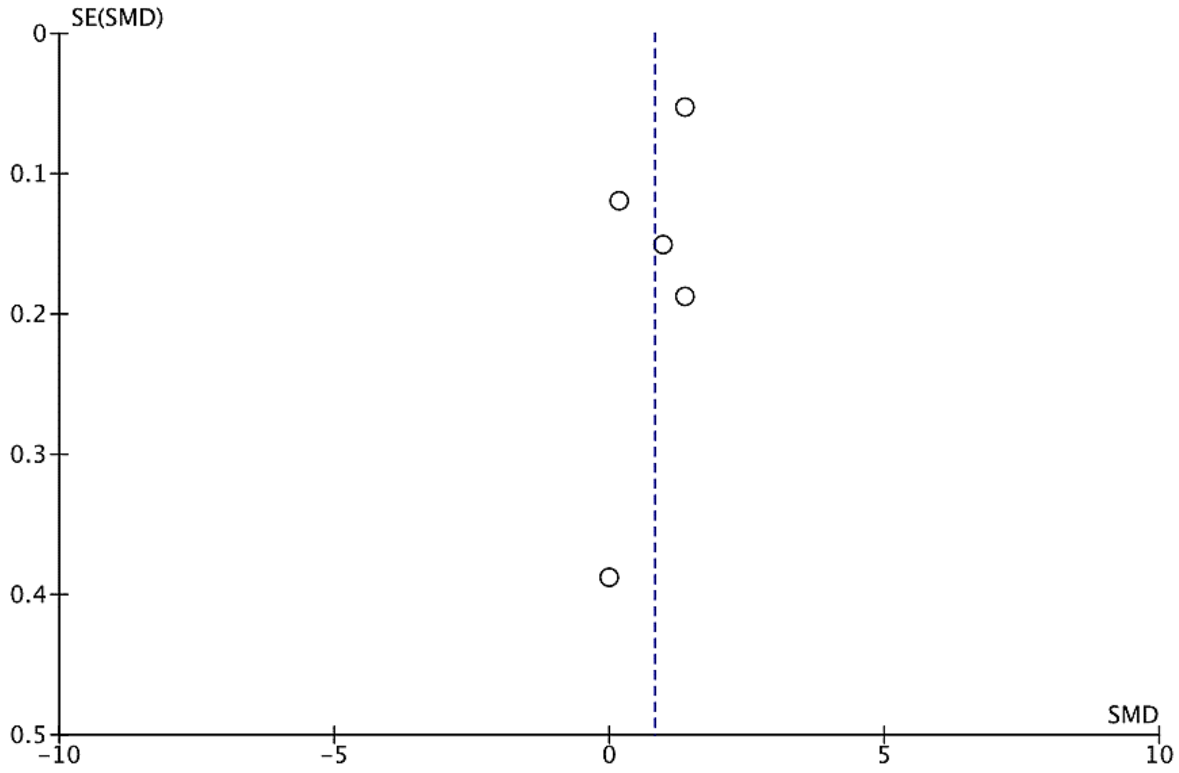

Figure S1. Funnel plot of comparison: final values, outcome: Adiponectin.

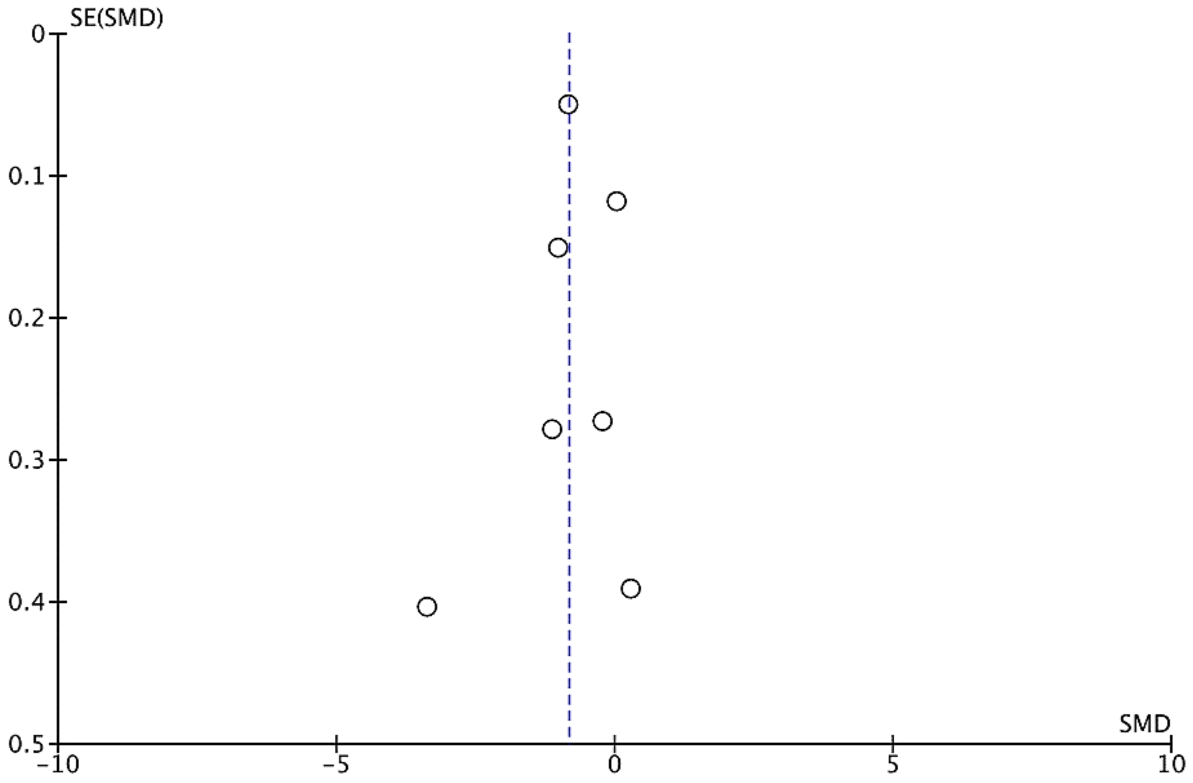

Figure S2. Funnel plot of comparison: final values, outcome: PCR.
